# Coupled circumferential and axial tension driven by actin and myosin influences *in vivo* axon diameter

**DOI:** 10.1038/s41598-017-13830-1

**Published:** 2017-10-27

**Authors:** Anthony Fan, Alireza Tofangchi, Mikhail Kandel, Gabriel Popescu, Taher Saif

**Affiliations:** 1 Department of Mechanical Science and Engineering, University of Illinois at Urbana-Champaign, Urbana, IL USA; 2 Department of Electrical and Computer Engineering, University of Illinois at Urbana-Champaign, Urbana, IL USA

## Abstract

It has long been known that neuronal axons are contractile. They actively maintain rest tension along the longitudinal direction both *in vitro* and *in vivo*. Here we show evidence that embryonic *drosophila* axons also actively maintain contractility/tension along the circumferential direction. We used confocal microscopy and spatial light interference microscopy to monitor axonal diameter along their length. We observed a decrease in diameter when microtubules are disrupted and an increase in diameter when actin filaments or myosin II are disrupted. Interestingly, active diameter reduction occurred consistently when axons were subjected to manipulations known to increase axial tension, suggesting that tension can be coupled in the axial and circumferential direction. This is further supported by the remarkably similar time constants for diameter reduction and rest tension increase of slackened axons. We infer that the actomyosin-driven circumferential contraction/hoop tension applies a squeezing force on the microtubule bundle of the axons. This hoop tension is balanced by the restoring force of the microtubule bundle. Therefore, axonal diameter increased when actin/myosin disrupting drugs relaxed the hoop tension and decreased when microtubule disrupting drug relaxed the restoring force. Circumferential tension thus can regulate axonal diameter and volume, as well as potentially microtubules alignment, inter-tubular spacing, and, by extension, axonal transport.

## Introduction

Neurons compute by integrating upstream signal inputs and propagating a single output. This signal integration and propagation requires the neuronal cell to take a specialized shape–usually in the form of many dendrites and an extended axon^1^. Microtubules (MT) has been thought to give axons their tubular structure^2^, and actin, in the form of a mesh network, was proposed to link the cytosolic MT to the axolemma^3^. The recent discovery of periodic subcortical actin rings and associated proteins in axons^4^ (and dendrites^5^) adds new insights to the cytoskeleton architecture. This highly regular actin network has been suggested^6,7^ and demonstrated^8^ to maintain structural integrity. How such is achieved, however, remains largely speculative.

The contractility of actin network, driven by myosin motors, is well-established to help shape cell morphology^9,10^. In axons, the acto-myosin machinery maintains an intrinsic axial tension *in vitro* ^11,12^ and *in vivo* ^13–15^. This tension can influence axonal processes such as growth^16–21^, synaptic plasticity^13,22^, and vesicle transport^23^ and release^24^. We speculate that tension could also exist in the circumferential direction, creating a hoop stress, as suggested by the ring-like actin network.

Here we use confocal microscopy and spatial light interference microscopy (SLIM)^25^ to infer circumferential tension by monitoring the diameter of single axons in embryonic *drosophila in vivo*. We report observations that suggest that this circumferential tension is coupled with the axial tension. It appears that axons regulate their diameter, and hence structure, through this coupled contractile tension.

## Materials and Methods

### Drosophila *Culture*

Transgenic embryonic *drosophila* (P(elav’-GAL4)[iii] P(UAS-gap::GFP, w+)[6iii]) with neuronal membranes tagged by green fluorescent protein (GFP) were used for the experiments. To harvest the embryo for dissection, flies were cultured on standard grape agar plates at room temperature (23 °C) for 18 hrs. The new embryos laid (0–18 hours old) were treated with 50% bleach for 1 minute for dechorionation, and subsequently rinsed with deionized water. We then visually inspected the embryos to select the ones with morphologies corresponding to those of stage 16. This protocol was adapted from Budnik *et al*.^26^.

### Dissection and Manipulation

Selected embryos were then transferred onto a double-sided tape (Scotch; 3 M, St. Paul, MN) attached to a large glass cover slip (12-545 H; Fisher Scientific, Hampton, NH). We positioned the embryo specifically with the dorsal side, where the CNS was, touching the tape. A glass needle (World Precision Instrument, Sarasota, FL), pulled by a micro-electrode puller (Sutter Instruments, Novota, CA), of 1 *μ*m tip size and a 20° taper was used to cut open the embryo from the posterior end to the anterior end. The fly, now free from all its cuticle, naturally adhered to the glass needle. We transferred it onto the cover slip glass surface, covered it with 500 mL of PBS (the volume is important for later drug concentration), and subsequently removed the inner organs until the body wall could be laid unrolled with the CNS exposed for further manipulations.

Intersegmental nerves, aCC motor neuron (MN) and RP2 MN, were identified from one section of the flat body wall. We gently excised the surrounding fat cells, muscle fibers, and nearby neuron while leaving the entire connection–CNS → aCC + RP2 → NMJ–intact.

A different glass needle of similar specifications was then brought close and made adhere to the muscle side of the NMJ using a piezoelectric micro-actuator (NanoPZ PZC200; Newport, Irvine, CA) that provided nanometer resolution.

### Pharmaceutical Drug

Cytochalasin D (50 *μ*g/mL) was used for the disruption of F-actin. Nocodazole (15 *μ*g/mL) and Colchicine (200 *μ*M) were used for the disruption of MT. Y-27632 (110 *μ*M) and ML-7 (225 *μ*M) were used to inhibit the ROCK and MLCK pathways respectively. All chemicals were purchased from Sigma-Aldrich (St. Louis, MO). DMSO was added as a solvent and was maintained at a final concentration of less than 1%, tested to have no observable effect^15^. All drugs were diluted in Ca^2+^/Mg^2+^ free PBS. Drugs were added to the samples 3 minutes before the onset of measurements.

### Microtubules Staining

The prepared embryos were extracted with BRB80-4mM EGTA-0.5% TX-100 (chemicals from Sigma, St Louis, MO) for 30 seconds to distinguish polymerized MT and fixed with 4% formaldehyde (28908; Fisher Scientific, Rockford, IL) for 15 minutes; blocked in 5% Bovine Serum Albumin (A9647; Sigma-Aldrich, St. Louis, MO) for 10 minutes; conjugated with mouse anti-*α*-tubulin (1:10; 12G10; DSHB, Iowa City, IA) for 60 minutes; tagged with Alexa 647 goat anti-mouse (1:200; A-21236; Fisher Scientific, Rockford, IL) for 30 minutes; and imaged with a scanning confocal microscope (LSM700; Carl Zeiss, Peabody, MA) afterwards. Samples were rinsed with PBST (PBS-0.1% Triton X-100) after each step. All steps were performed at room temperature.

### Confocal Imaging

The embryos were inspected using a confocal microscope (LSM700; Zeiss, Oberkochen, Germany). The 488-nm laser was used to excite the GFP and emission light with wavelength larger than 488 nm was collected. Pinhole size was set to 1 a.u. A z-stack distance of 0.41 *μ*m was maintained. A 40x/1.3 objective was used. We note that the number of images (ranges from 15–50) for any axon depends on its degree of out of plane tilt. We discarded samples with too much tilting that needed more than 50 images in the stack. The scan speed was sometimes adjusted so that an image stack could be obtained in around 80 seconds. The settings were kept consistent across all independent experiments. Image stacks were taken every 5 minutes. We found that this minimized photobleaching while allowing images to have adequate spatial and temporal resolution for our analysis.

### Image Analysis

The collected images were post-processed first using ImageJ (U.S. National Institutes of Health, Bethesda, MD). The z-stacks were collapsed to single images by maximum intensity projection. The images were cropped to only the axon of interest which were then transferred to MATLAB (MathWorks, Natick, MA). A batch-enabled script was used to detect the edge of the axons, and subsequently performed diameter and area calculations (Fig. S1). The edge was traced by fitting a Gaussian profile to the intensity profile along the x-axis for every y (axial direction of axon). The 2 locations with maximum slope ($\frac{{d}^{2}I(x)}{d{x}^{2}}=0$) were reported for each y-coordinate. Connecting those points for all y-coordinates provided a discretized edge with axial resolution of a single pixel and lateral resolution of at least half a pixel. 3D images were generated by ImageJ to ensure that the distance between the 2 projected edges was representative of the diameter (Movie 1). Using the edge values, average diameters were computed over the same visible axonal length for each condition among different time points.

### Spatial Light Interference Microscopy (SLIM)

SLIM was also used to examine the diameter and axonal mass transport. The sample preparation is similar to that previously described with slight modification to facilitate phase imaging. Specifically, we added a top cover slip above a PDMS spacer to seal off the sample and prevent evaporation. In addition, the space between the two cover slips (one supporting the embryo and the top cover) was filled with PBS. This offered flat interfaces for PBS to avoid any bias in the phase values during light transmission. Because of the top cover slip, no stretching manipulation was done to the samples imaged with SLIM.

In short, SLIM is a quantitative phase imaging^27^ technique that measures optical path length shifts in a phase contrast geometry^25^. In our implementation (CellVista SLIM, Phi Optics, Inc.), the pupil plane of a Zeiss Z1 is projected onto a spatial light modulator (SLM) that is then cycled in *π*/2 increments, essentially modulating the phase ring internal to the objective. Four such images are combined^28^ to produce a full-field phase-map, with a radian value at each pixel. All SLIM images used in this work were acquired with a 63x/1.4 objective (PN 420781-9910) and an sCMOS camera (Andor Zyla 5.5).

Each stack was used to reconstruct a single image to facilitate analysis (Fig. S2). The stack was first cropped to a region that only the axon of interest was visualized. Then, for each 5-pixel-times-width region along the axial direction of the axon, we found the stack plane with maximum gradient magnitude and defined the plane as the focal plane. The obtained plane numbers were then smoothed using the robust linear method and subsequently interpolated for every pixel along the longitudinal direction of the axon. These values were then rounded and used to obtained a reconstructed image from the image stack.

To evaluate diameter, we made use of the negative phase region at the boundaries of the axon (Fig. S3). Similar to the Gaussian fitting previously described, we obtained a profile, this time in phase values, for every pixel along the axial direction. We smoothed the profile and computed its difference function. The 2 maximum peaks (locations with maximum slope) in the difference function bound by the negative regions were defined as the boundary.

### Data Availability

The datasets generated during and/or analysed during the current study are available from the corresponding author on reasonable request.

## Results

**Figure 1:**
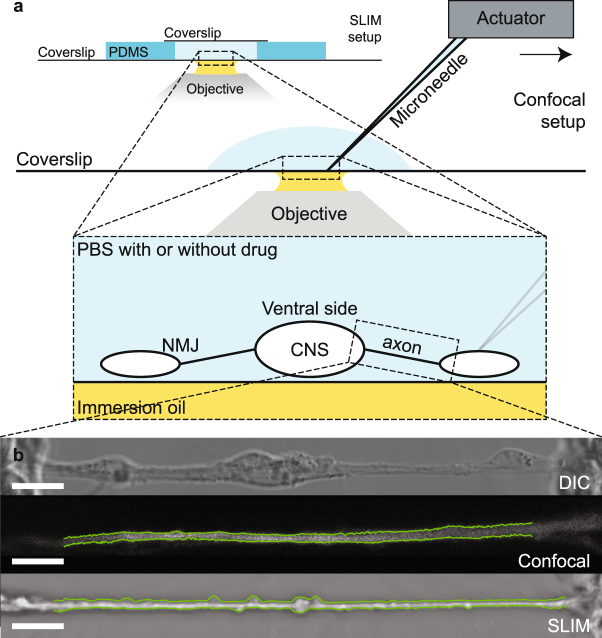
Sample preparation and representative images. (**a**) Cartoon schematic of the experimental setup with both imaging methods. Note that no stretch experiment was carried out in the SLIM set-up due to the top cover slip; stretching manipulations were done exclusively using the confocal setup. (**b**) (top) DIC image of an axon before cleaning is completed. (center) Maximum-intensity-projected confocal image of a cleaned axon. Green lines labeled the boundaries determined by the analysis algorithm. (bottom) Reconstructed SLIM image of another cleaned axon. Green lines labeled the boundaries determined by the analysis algorithm. All scale bars at 10 microns.

**Figure 2:**
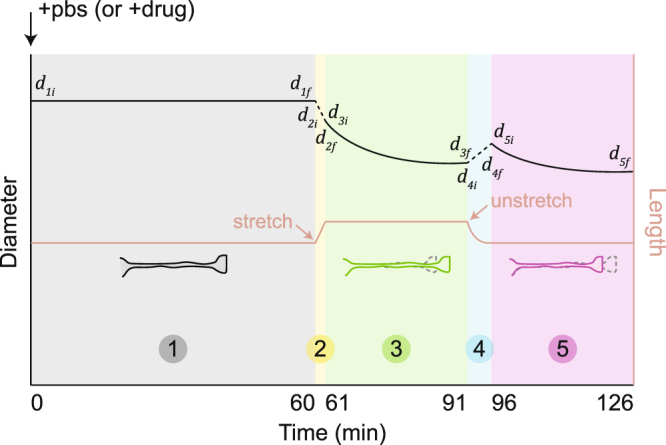
Continuous sample plot of diameter data and stretching manipulations. Phase 1: PBS (or drug) treatment of 60 minutes (Fig. 3). Phase 2: Stretch to 15–25% strain in less than 1 minute. Drop in diameter due to volume conservation (Fig. 4b). Diameter is not monitored. Phase 3: Stretch is held and diameter is traced for 30 minutes (Fig. 4c). Phase 4: Unstretch the sample to its original configuration. Axon would first buckle and then contract to a straight configuration again in 5 minutes. Gain in diameter due to volume conservation (Fig. 5c). Diameter is not monitored. Phase 5: Axon rebuilds its rest tension and diameter is traced for another 30 minutes (Fig. 5b). Experiements (Figs 4e and 5a in main text) not subjected to phase 1 follow identical phases 2–5.

In order to image axon diameter continuously, invasive surgery was performed on *drosophila* embryo to clean the tissues (including glial cells^15^) surrounding the axon until single axons could be observed. The neural membrane was genetically labelled with GFP for visualization purposes. Two imaging methods were used–confocal microscopy and SLIM. The preparations were similar with SLIM samples having a top cover slip to facilitate phase imaging (Fig. 1a). Fluorescence signal from the neural-membrane-bound GFP provided contrast in the confocal system, while phase changes provided contrast in SLIM. Methods unique to each imaging modality were used to (1) reconstruct an image stack into a single image, and (2) compute the diameter (and average phase for SLIM) from the reconstructed image (Fig. 1b). See materials and methods for detailed description. Axons were subjected to a series of stretching manipulations as depicted in Fig. 2.

### Actin maintains a circumferential tension resisted by microtubules

**Figure 3:**
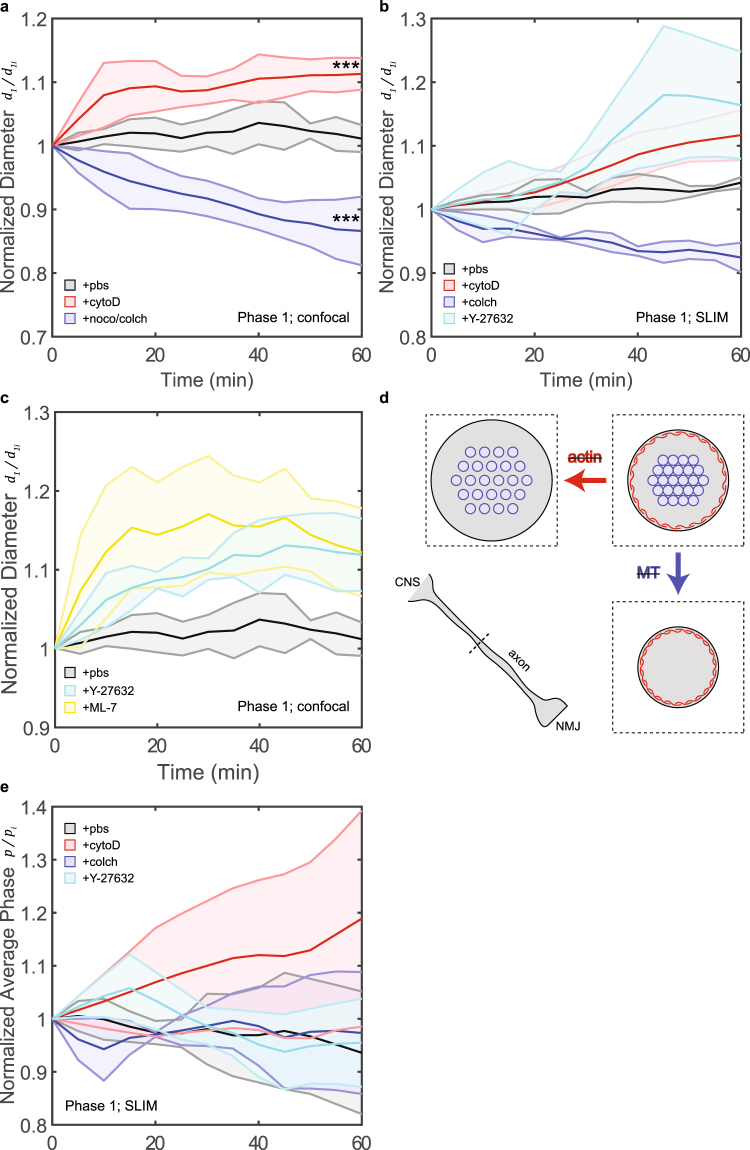
Diameter changes under drug treatment. (**a**) Confocal and (**b**) SLIM measurements of average diameter over time of axons treated with PBS (grey), cytoD (red), noco/colch (blue), and Y-27632 (cyan). (**c**) Confocal measurements of average diameter over time of axons treated with PBS (grey), Y-27632 (cyan), and ML-7 (yellow). (**d**) Cartoon schematic of the proposed explanation. Figures (in dotted boxes) depict cross-section, as indicated by the dotted line in the bottom left figure, of axons under different conditions. Red network represents actin filaments, and blue circles indicates MT. When actin filaments, and therefore circumferential tension, are disrupted, a dilation in diameter (and volume) occurs. This can potentially cause MT to become less compact. When MT are disrupted, actin filaments have less resistance to compact resulting in a smaller diameter (and volume). (**e**) Average phase measured by SLIM of axons treated with pbs (grey), cytoD (red), colch (blue), and Y-27632 (cyan). The average density of the cytoplasm and the cytoskeletal components increase with time with disruption of actin, but not with MT. All shaded regions indicate error bar in standard deviation. Unpaired two-sample t-test used to obtain p-values.

Drugs with known specificity to actin filaments (cytochalasin D, cytoD) and MT (nocodazole and colchicine, noco/colch) were applied to the preparations 3 minutes before imaging commenced. The preparations were monitored under the two imaging modalities for 60 minutes (phase 1 in Fig. 2). We measure initial diameter at ${d}_{1i}=2.34\pm 0.44$ *μm* (confocal, N = 6) & $3.31\pm 0.49$ *μm* (SLIM, N = 6). This corresponds to at least 20 pixels across the width of an axon (Fig. S4). We estimated that our gaussian fitting measurements should be accurate for at least 0.5 pixels. This gives a resolution of around 2.5%. The diameter changes were largely consistent between the two modalities (confocal imaging and SLIM). Disrupting actin filaments (Fig. 3a and b, red; Movie 2) led to an increase in average diameter (confocal: ${d}_{1f}/{d}_{1i}=1.11\pm 0.02$, $N=8$; SLIM: ${d}_{1f}/{d}_{1i}=1.12\pm 0.04$, $N=3$), and disrupting MT (Fig. 3a and b, blue) led to a decrease in average diameter after 60 minutes of drug treatment (confocal: ${d}_{1f}/{d}_{1i}=0.87\pm 0.05$, $N=6$; SLIM: ${d}_{1f}/{d}_{1i}=0.92\pm 0.02$, $N=3$). Control cases with PBS incubation (Fig. 3a and b, grey) showed a small increase (confocal: ${d}_{1f}/{d}_{1i}=1.01\pm 0.02$, $N=6$; SLIM: ${d}_{1f}/{d}_{1i}=1.04\pm 0.01$, $N=3$) and slight fluctuations within our estimated accuracy of 2.5%. Spatial examination also revealed that diameter increase caused by actin disruption was consistent along the entire length of each axon (Fig. S5).

To test whether contractile forces are involved in diameter regulation, we treated the axons with myosin II disrupting drugs, Y-27632 and ML-7. These drugs were found to reduce contractility of axons along the longitudinal direction^15^. We observed diameter increase when axons were treated with Y-27632 (confocal: ${d}_{1f}/{d}_{1i}=1.12\pm 0.05$, $N=3$; SLIM: ${d}_{1f}/{d}_{1i}=1.16\pm 0.08$, $N=3$) and ML-7 (${d}_{1f}/{d}_{1i}=1.12\pm 0.06$, $N=3$) respectively (Fig. 3b and c). This implies that relaxation of tension results in increase in diameter.

Although the final magnitude of diameter increase (under cytoD or Y-27632 treatment) is similar between the 2 modalities, the time constant is not. We believe this can be attributed to the imaging and analysis methods used. In confocal imaging, the GFP signal comes from a membrane bound protein expressed only in neurons. While in SLIM, the contrast is derived from the content of the axons. Furthermore, we used a curve fitting algorithm in analyzing the data from confocal imaging to minimize noise, and such was difficult to realize in SLIM because of the many local intensity peaks. Previous work suggests that actin filaments are sensitive to cytoD treamtent in less than 10 minutes^29^, which is what we observed in confocal imaging.

These observations can be explained by contractility of actomyosin machinery along the circumferential direction of axons (Fig. 3d). When actin filaments/myosin II are disrupted, the circumferential tension is diminished. This leads to an imbalance of cell pressure and membrane tension, and, as a result, an inflation in diameter (and volume). On the other hand, when MT are disrupted, the polarity of the imbalance switches since there are now less restoring force against contractile actin leading to reduction in diameter. This proposition is interestingly similar to the case of axial axonal tension^15^, leading to our speculation that tension could be coupled in the axial and circumferential directions.

SLIM imaging revealed that the average phase (*p*) increased (${p}_{f}/{p}_{i}=1.19\pm 0.20$, $N=3$) when actin was disrupted (Fig. 3e, red). Average phase remained unchanged (colch: ${p}_{f}/{p}_{i}=0.97\pm 0.11$, $N=3$; Y-27632: ${p}_{f}/{p}_{i}=0.95\pm 0.08$, $N=3$) upon MT disruption (Fig. 3e, blue) or Y-27632 treatment (Fig. 3e, cyan). As phase correlates with mass density^30^, this suggests that the content that left upon MT disruption had similar density on average to the remaining content in the axon, while the new mass that had come in due to the inflation in diameter upon actin disruption was denser on average. Increase in diameter caused by Y-27632 did not lead to an influx of denser mass. This suggests that an inflation in diameter alone is not sufficient to cause the influx of denser mass; actin depolymerization is necessary.

The increase in phase upon actin disruption led us to investigate any side effects that CytoD itself might have caused. Upon a subsequent washout, axons that were treated with CytoD revert back to their original diameter (Fig. S4b). This suggests that CytoD did not cause any detrimental effect on axons permanently.

We further perform an analysis to understand the nature of the heavier mass coming in upon diameter inflation. We compared the normalized phase distribution of axons before and after cytoD treatment and identified a continuous range that led to the reported increase in average phase (Fig. S6a). The pixels that fell within the identified range show structures that resemble vesicles (Fig. S6b).

### Axonal volume is conserved under fast stretch

To further examine whether axial and circumferential tension are coupled, we applied extra tension to the axon by stretching it (15–25% strain) from the NMJ side using a micro-pipette tip connected to a piezo-actuator (phase 2 in Fig. 2).

During initial fast stretch, mass flow in or out of axons is limited and volume should be conserved. We verified such conservation of volume during initial stretching by comparing theoretical diameter ratios to experimental values. Given the definition of strain ($\varepsilon$) as $\frac{{\rm{\Delta }}l}{l}$, the stretch ratio ($\lambda$) is defined as:

1 $$\lambda =\frac{l}{{l}_{0}}=\frac{{l}_{0}+{\rm{\Delta }}l}{{l}_{0}}\mathrm{=1}+\varepsilon .$$

The volume ratio for an axon modeled as a cylindrical rod is then:

2 $$\frac{{V}_{2f}}{{V}_{2i}}=\frac{{d}_{2f}^{2}{l}_{2f}}{{d}_{2i}^{2}{l}_{2i}}=\frac{{d}_{2f}^{2}}{{d}_{2i}^{2}}\lambda .$$

If volume is conserved, then volume ratio $(\frac{{V}_{2f}}{{V}_{2i}})$ is 1. The diameter ratio (${d}_{2f}/{d}_{2i}$) is then:

3 $$\frac{{d}_{2f}}{{d}_{2i}}=\frac{1}{\sqrt{\lambda }}={\mathrm{(1}+\varepsilon )}^{-\frac{1}{2}}.$$

**Figure 4:**
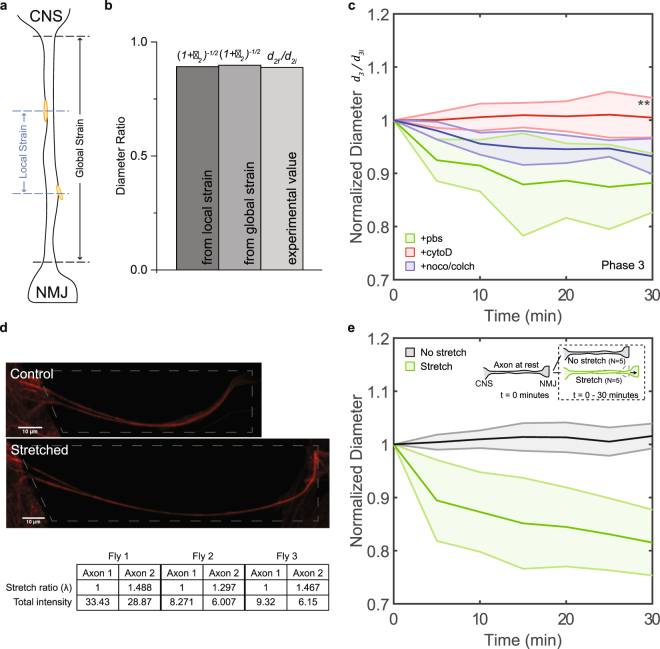
Diameter reduction driven by actin filaments upon a sustained stretch. (**a**) Cartoon schematic describing the concept of local strain (between 2 visible markers) and global strain (between 2 ends). (**b**) Diameter ratios as calculated from local and global strain and observed from experiment. (**c**) Diameter of axons held at prescribed stretch (15–25%) under PBS (green), cytoD (red) and noco/colch treatment (blue). (**d**) Two neighboring axons with similar length are cleaned per embryo. One is stretched (bottom) and the other is left unperturbed (top). Stretch is held for 30 minutes, after which MT staining is performed. The total fluorescence intensity (intensity sum over all pixels within the bounded 3D region) after background subtraction, which measures the amount of MT in each axon, is quantified and compared. Stretch ratio defined in Eq. 1. Total intensity shown in arbitrary unit. (**e**) Axons imaged immediately after embryo surgery with (green) or without (grey) stretch applied. Inset cartoon depicts the two processes. All shaded regions indicate error bar in standard deviation. Unpaired two-sample t-test used to obtain p-values.

Using Eq. 3, we can test volume conservation by comparing diameter and strain. Two strain measurements were used. One, termed global strain, traced the two ends of the axon. The other, termed local strain, traced two clearly identifiable points along the axon (Fig. 4a). The results for one axon are shown in Fig. 4b. We find, the diameter ratio is almost equal to ${\mathrm{(1}+\varepsilon )}^{-\frac{1}{2}}$ for both local and global strains. Thus Eq. 3 is satisfied experimentally, implying volume conservation. The close agreement between local strain and global strain suggests that the axonal strain is uniform along its length. In addition, it suggests that mechanical elastic properties of the axon is also uniform along its length. Data from three samples are shown in Table 1.

**Table 1:** Comparison of local strain, global strain and ${{d}}_{2{f}}/{{d}}_{2{i}}$ in Phase 2.

**Sample**	**Global Strain**	${(1+{\varepsilon }_{2,glo})}^{-\frac{1}{2}}$	**Local Strain**	${(1+{\varepsilon }_{\mathrm{2,}loc})}^{-\frac{1}{2}}$	${d}_{2f}/{d}_{2i}$
1	25.8%	0.891	24.0%	0.898	0.888
2	21.2%	0.908	19.2%	0.916	0.912
3	13.1%	0.940	13.3%	0.939	0.945

### Circumferential tension from actin filaments reduces axon diameter overtime when a mechanical stretch is held

We monitor diameter changes for 30 minutes with the stretch held fixed (phase 3 in Fig. 2). The diameter continued to decrease (${d}_{3f}/{d}_{3i}=0.88\pm 0.06$, $N=6$) for axons treated with PBS (Fig. 4c, green). This trend was not observed (${d}_{3f}/{d}_{3i}=1.00\pm 0.04$, $N=6$) in axons with actin filaments disrupted (Fig. 4c, red). A smaller decrease was observed (${d}_{3f}/{d}_{3i}=0.93\pm 0.03$, $N=6$) in axons with MT disrupted (Fig. 4c, blue). The reduction of diameter upon a sustained stretch, again, occurred along the entire length (Fig. S4). Thus volume of the axon under sustained stretch reduced in PBS and with MT disruption, but not with actin disruption.

Previous studies have shown that upon fast stretching, axial tension increases instantaneously, and then slowly relaxes (due to viscoelastic response to the applied tension) to a state that is more tensed (around 3-fold at 60% strain) than that prior to any stretch (rest tension)^31^; i.e., axial tension increases after stretch. This, together with our results that stretching led to diameter reduction (circumferential tension increases) driven by actin filaments, again suggests that axial tension and circumferential tension could be coupled.

We hypothesized that the actomyosin-driven reduction in diameter under sustained stretch was mainly due to the breakage of MT upon sudden mechanical stretch^32^, creating space for actin filaments to contract further. This is supported by the similar diameter reduction in MT-disrupted unstretched axons in phase 1 and untreated stretched axons in phase 3 (Fig. 3a, blue vs. Figure 4c, green). In addition, the smaller reduction in diameter in stretched axons with MT previously disrupted (Fig. 4c, blue) compared to untreated ones (Fig. 4c, green) further supports the argument; in these cases, MT had been disrupted and already resulted in a diameter reduction in phase 1. To test this hypothesis, staining experiments were performed to show the reduction of polymerized tubulin upon stretching (Fig. 4d).

Furthermore, as a control against prolonged saline incubation during phase 1, we performed stretching manipulations on a few axons immediately after embryo surgery. The observed diameter reduction showed no significant differences compared to that after 60 minutes of PBS incubation (Fig. 4c and e, green). Diameter reduction was not observed in axons not subjected to any stretch (Fig. 4e, grey) similar to results in Fig. 3a and b.

### Circumferential tension from actin filaments reduces axon diameter when axon is returned to initial configuration

**Figure 5:**
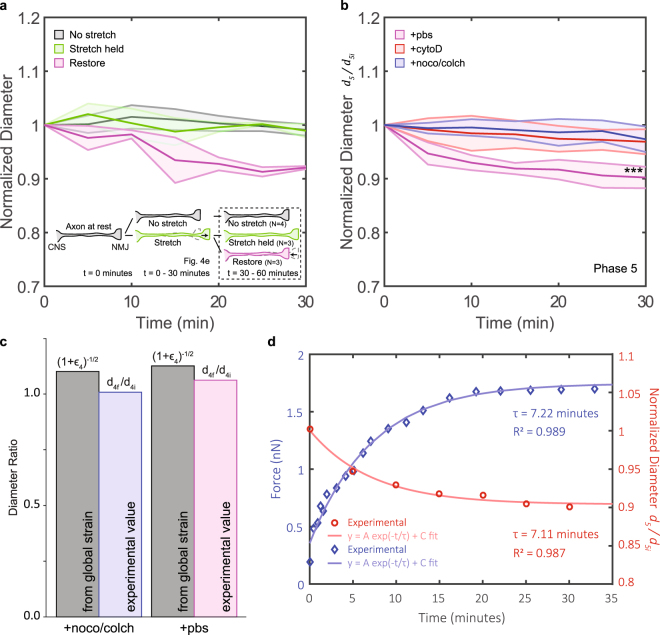
Actin-driven diameter reduction upon return to initial configuration. (**a**) Diameter normalized to the initial value of axons with no stretch (grey), stretch held (green), and stretch restored (magenta). The three processes are described in the inset cartoon: Axons are at rest initially. Some axons are stretched (15–25%) and the stretch is held for 30 minutes. A few axons are then returned to the original configuration. All axons are then monitored for another 30 minutes. (**b**) Diameter of restored axons subjected to PBS (magenta), cytoD (red), and noco/colch (blue) treatment. (**c**) Diameter ratios (similar to those in Fig. 4b) during phase 4 plotted for 2 axons treated with noco/colch and pbs respectively. The axons are selected for similar ${\mathrm{(1}+{\varepsilon }_{4})}^{-\frac{1}{2}}$. See also Table 2. (**d**) Axial tension of a restored axon in blue (data previously published in Rajagopalan *et al*.^31^). Normalized diameter from 5c plotted in red. Both data sets are fitted to an exponential equation (form given in the plot). ${R}^{2}$ and $\tau$ values are displayed for comparison purposes. Circumferential, as inferred from diameter reduction, and axial tension have similar temporal dynamics. All shaded regions indicate error bar in standard deviation. Unpaired two-sample t-test used to obtain p-values.

After the stretch was held for 30 minutes, some axons were brought back to their initial configuration. We called this process “unstretch” (phase 4 in Fig. 2). The axons first buckled when subjected to the unstretch process. They then slowly contracted to straighten again. Note that axons treated with cytoD do not fully contract, as also observed in other studies^15^, because actin-myosin contractility is hampered. For all instances, we waited 5 minutes after which their length did not visibly change. Axons left stretched served as control (Fig. 5a).

We then continued to monitor the diameter for another 30 minutes (phase 5 in Fig. 2). We called this phase “restore” for those axons returned to their taut, straight configuration. Previous studies have shown that axons build up significant tension^31^ that is actin-myosin dependent^15^ during this phase. We observed diameter reduction in restored axons (${d}_{5f}/{d}_{5i}=0.90\pm 0.02$, $N=6$), regardless of whether phase 1 had previously been executed (Fig. 5a and b, magenta; Movie 3). Diameter did not change in control cases where axons were either never stretched (Fig. 5a, grey) or remained stretched without being restored (Fig. 5a, green), as explained in the inset of Fig. 5a. Reduction in diameter was significantly hampered (${d}_{5f}/{d}_{5i}=0.97\pm 0.02$, $N=5$) in axons with actin filaments distupted (Fig. 5b, red).

However, axons with MT disrupted (Fig. 5b, blue) showed no diameter reduction (${d}_{5f}/{d}_{5i}=0.97\pm 0.02$, $N=5$). We expected higher reduction in diameter since MT disruption results in higher longitudinal tension in axons^12,33^, and hence higher circumferential tension if there is coupling. The paradox can be resolved by considering phase 4, in which we waited 5 minutes for the axons to straighten after unstretch. It is known that force-free slackened axons with MT disruption straighten 3 times faster compared to axons with MT intact^15^. Volume conservation requires that soon after straightening diameter ratio increased to ${\lambda }^{-\mathrm{1/2}}$. After straightening, tension built up fast in MT-disrupted axons. Diameter reduced and arrived at a steady value before phase 5 began. This explains our experimental finding of ${d}_{4f}/{d}_{4i} < {\lambda }^{-\frac{1}{2}}$ (Table 2 & Fig. 5d, blue). Axons not treated with any drugs also straightened before phase 5 commenced. And diameter reduction (relative to the diameter from volume conservation calculations) in phase 4 was also observed in these axons (Table 2 and Fig. 5d, magenta); the reduction however continued in phase 5. This supports the argument that circumferential tension is coupled to axial tension: disrupting MT has been shown to speed up axial contraction and increase axial tension^12,15,33^, and here from our measurements we infer that MT disruption can lead to faster circumferential contraction.

**Table 2:** Comparison of global strain and ${{d}}_{4{f}}/{{d}}_{4{i}}$ in Phase 4.

Sample	Treatment	Global Strain	${({\bf{1}}+{{\boldsymbol{\varepsilon }}}_{{\bf{4}}})}^{-\frac{{\bf{1}}}{{\bf{2}}}}$	${{\boldsymbol{d}}}_{{\bf{4}}{\boldsymbol{f}}}/{{\boldsymbol{d}}}_{{\bf{4}}{\boldsymbol{i}}}$
1	+noco/colch	−10.9%	1.059	1.019
2	+noco/colch	−14.3%	1.080	0.973
3	+noco/colch	−17.5%	1.101	1.008
4	+pbs	−21.2%	1.126	1.062
5	+pbs	−24.9%	1.153	1.052
6	+pbs	−28.4%	1.182	1.082

We compared diameter reduction as a function of time with the evolution of tension in slackened axon measured previously^31^. Both data sets fitted well to an exponential curve in the form of $A{e}^{-t/\tau }+C$ (Fig. 5d). Time constants (axial tension: 7.22 minutes; diameter: 7.11 minutes) were extracted from the fits. The time constant for tension development and diameter reduction are comparable, implying a common mechanism generating axial tension and reduction of diameter. This further supports that axial and circumferential tension could be coupled. Note that these time constants are greater than those observed in length shortening of slackened axons^15^, because axons continue to apply tension after becoming taut. This does not change the length, but can be captured using a force sensor^31^.

## Discussion

**Figure 6:**
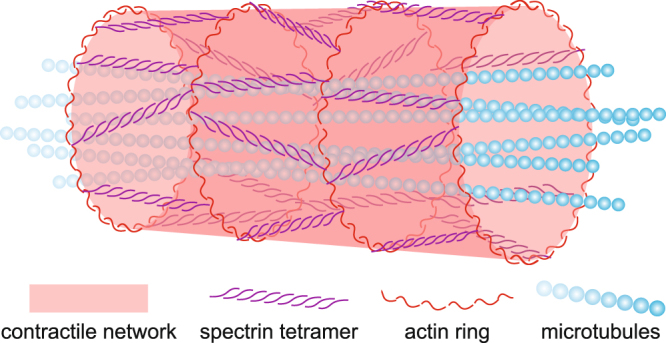
Cartoon illustration of the proposed descriptive model. Cortical actin and spectrin form a contractile network. Cytosolic MT provides structural support and act against the axial and circumferential tension generated by the contractile network.

In this paper we explored the biophysical factors that influence axonal diameter in motor neurons of embryonic *drosophila*. We used two independent methods, confocal microscopy and SLIM imaging, to measure axonal diameter. We demonstrated earlier that after synaptogenesis axons tends to contract and hence develop tension along the longitudinal direction. Actin and myosin II are involved in generating the longitudinal tension^15^. Here we show that acto-myosin machinery is involved in generating circumferential tension as well along the entire length of the axon. This tension originates from the contractility of cortical actin, which in turn applies a compressive force on the MT. The force balance between cortical actin and microtubule results in an equilibrium diameter of the axon (Fig. 6). Thus, when MT are disrupted, diameter decreases; when actin is disrupted or myosin II is inhibited, diameter increases. We further show that longitudinal and circumferential tension are coupled. They share similar time constants of evolution; i.e., times to generate longitudinal tension and to contract the diameter to their respective steady values are similar. This suggests a common mechanistic origin for both. However, the detailed cytoskeletal architecture that gives rise to the circumferential contractility of embryonic axons remains to be resolved.

Circumferential contractile forces are found in multiple biological processes. For example, actomyosin rings are critical for wound healing^34,35^ and cytokinesis^36,37^. In both cases, they exert a circumferential tension to do work and drive the dynamics of the processes. Notably, these other rings are much larger in size and can span several cells; they are also more dynamic than those observed in axons^38^. Furthermore, the ring structure of actin has not yet been observed in myelinated axons, except that at the nodes of Ranvier^5^. *drosophila* axons are not myelinated, but glial cells still form an insulating layer around them as early as stage 15^39^. This limits the ability to super-resolve structure of actin (and other proteins) because of multiple scattering events^5^. Nonetheless, most evidence support the existence of actin ring in peripheral axons. It has been shown that axonal diameter increases when adducin^8^, an actin-capping protein that is thought to link the actin and spectrin in the periodic membrane skeleton, is disrupted; it is unclear though how adducin can help regulate diameter. The present work provides new insights to the mechanism by which actomyosin network can maintain structural integrity and regulate shape of axons–through the coupled axial and circumferential tension.

It is important to note that we used change of diameter of axons and the effect of force relaxing drugs to infer circumferential tension, given that force and deformation (change of diameter) are related through the cytoskeletal elasticity of the axons. Since these material properties are not known, we cannot quantify the circumferential forces. There is no direct means available to measure circumferential tension. The nano-Newton force probe used to measure longitudinal force of the axons^13^ could not be used to measure circumferential force. However, our data supports a model where longitudinal and circumferential tensions are coupled (Table 3), although the precise nature of this coupling remains elusive.

**Table 3:** Comparison of axial tension and circumferential tension inferred from diameter changes!

Treatment	Stretch state	Axial tension	Reference	Diameter	Cir. tension
PBS	No	No change	15,31	No change	No change
cytoD	No	↓	12,33	↑	↓
noco/colch	No	↑	12,33	↓	↑
PBS	Stretch	↑	31	↓	↑
cytoD	Stretch	?		No change	No change
noco/colch	Stretch	?		↓	↑
PBS	Restore	↑	15,31	↓	↑
cytoD	Restore	No change	15	No change	No change
noco/colch	Restore	↑	15	↓	↑

Our SLIM experiments demonstrated that mass displaced during diameter reduction (following MT disruption) did not change the density of the axon. Images from electron microscopy show that axons are MT-rich^3^. These 2 observations together suggest that the displaced mass is composed of depolymerized MT and their associated proteins. Mass that entered the axon during diameter dilation after actin disruption, on the other hand, had higher density and resulted in new structures with vesicle-like morphology based on our phase histogram analysis. One plausible explanation is that these new structures were originally vesicles scaffolded by an actin-rich network in the presynaptic terminal^40^, which upon actin disruption, are released into the axon. Yet, we cannot exclude the possibility of vesicles/cargo from other sources. Channels gating and vesicles fusion can also regulate mass; it has been shown that membrane tension provided by actin dynamics is a key factor in these processes^35,41^. We, however, did not observe exocytosis/endocytosis events in our experiments, which could be due to insufficient spatial-temporal resolution.

The reduction in diameter under stretch might seem counterintuitive to normal development, since one would expect the axon to increase its diameter during development. In fact, several *in vitro* experiments have shown that axons, when towed, would grow both axially and laterally^19,42^; the growth is dependent on new MT formation^43^. In these studies, however, the stretch is imposed slowly and gradually throughout the course of the experiment, while in our case the time from no stretch to full stretch (120% of original length) is usually in less than a minute. This seems to suggest that stretching with different strain rates can result in different MT dynamics; i.e., when stretch is applied at a higher rate than the polymerization process, MT can break and subsequently disassemble^32^, which occurs at a faster time scale^44^.

Axonal diameter is a physiologically relevant parameter, since conduction speed of action potential along the axons scales with the diameter for both healthy and pathological neurons^45^. Nerve atrophy results in reduction of axonal diameter and a corresponding reduction in conduction speed. Hence, significant effort has been directed to measuring axonal and nerve diameter using multiple methods including electron microscopy^46^, and diffusion MRI^47^. However, how axons maintain their diameter has gained limited attention. This work reveals a biophysical mechanism that maintains the diameter.

## Electronic supplementary material

3D maximum intensity projection of an exposed axon

An axon with increasing diameter when treated with cytoD during phase 1

An axon with decreasing diameter during the restore phase 5

Supplementary Material
