# Supplementary material for: Coupled circumferential and axial tension driven by actin and myosin influences in vivo axon diameter
Source: Sci Rep. 2017 Oct 27;7:14188. doi: 10.1038/s41598-017-13830-1 (PMC5660205; doi:10.1038/s41598-017-13830-1)
Supplement: Supplementary file 4 — Supplementary Material [file 41598_2017_13830_MOESM4_ESM.pdf]

Coupled circumferential and axial tension driven by actin and myosin influences *in vivo* axon diameter
Supplementary Material

Anthony Fan[1], Alireza Tofangchi[1], Mikhail Kandel[2], Gabriel Popescu[2], and Taher Saif*[1]

[1] *Department of Mechanical Science and Engineering, University of Illinois at Urbana-Champaign, Urbana, IL, USA*
[2] *Department of Electrical and Computer Engineering, University of Illinois at Urbana-Champaign, Urbana, IL, USA*

September 24, 2017

*corresponding author: saif@illinois.edu

Contents

Supplementary Figure **3**
Supplementary Figure S1 . . . 3
Supplementary Figure S2 . . . 4
Supplementary Figure S3 . . . 5
Supplementary Figure S4 . . . 6
Supplementary Figure S5 . . . 7
Supplementary Figure S6 . . . 8

Supplementary Note **9**
Robustness of average diameter $\langle d \rangle$ . . . 9

Supplementary Figure

Supplementary Figure S1

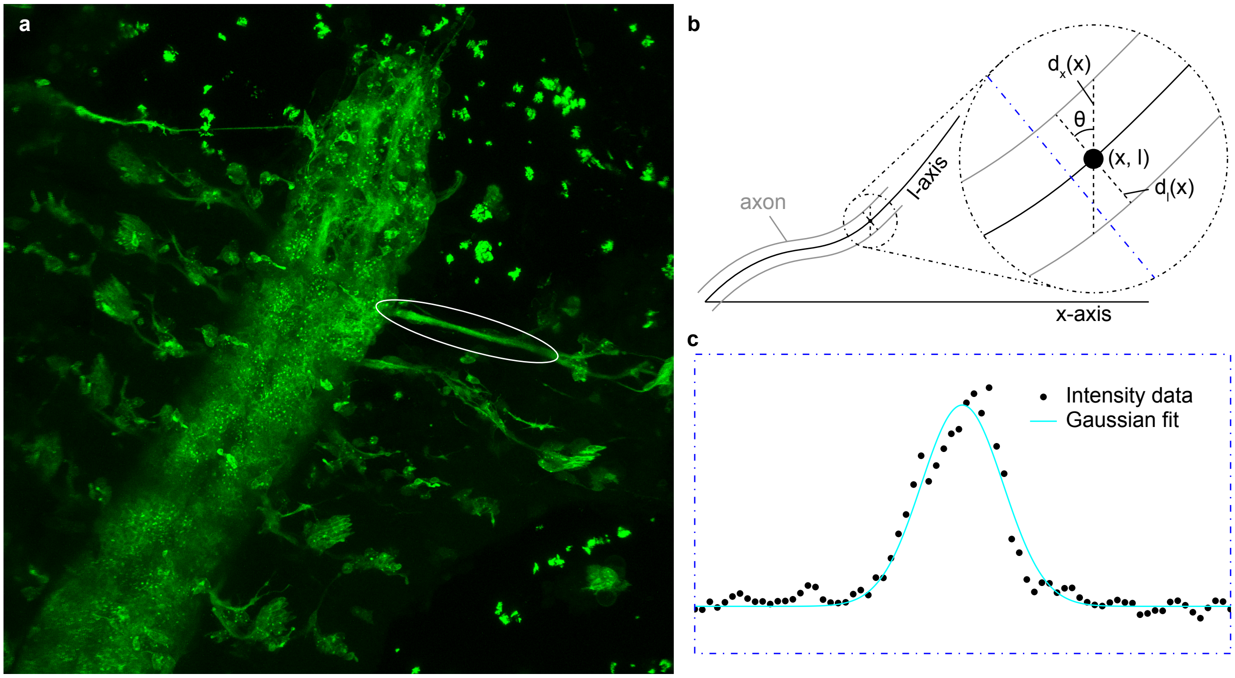

Figure S1: Approach to diameter calculation. (a) Maximum intensity projected fluorescence image of a dissected fly embryo. Signals are from neural membrane bound GFP. The white ellipse indicates a cleaned axon. (b) Schematics of an axon that has move away from its original axis (from x-axis to l-axis). Values of average diameter computed along the 2 axes separately are different by a factor of $cos\theta$. Detailed descriptions in supplementary notes. (c) Gaussian curve (G) fitted to maximum fluorescence intensity data along the cross-section (blue dotted line in b) of a randomly-selected axon. Points where $G'' = 0$ is defined as the boundary.

Supplementary Figure S2

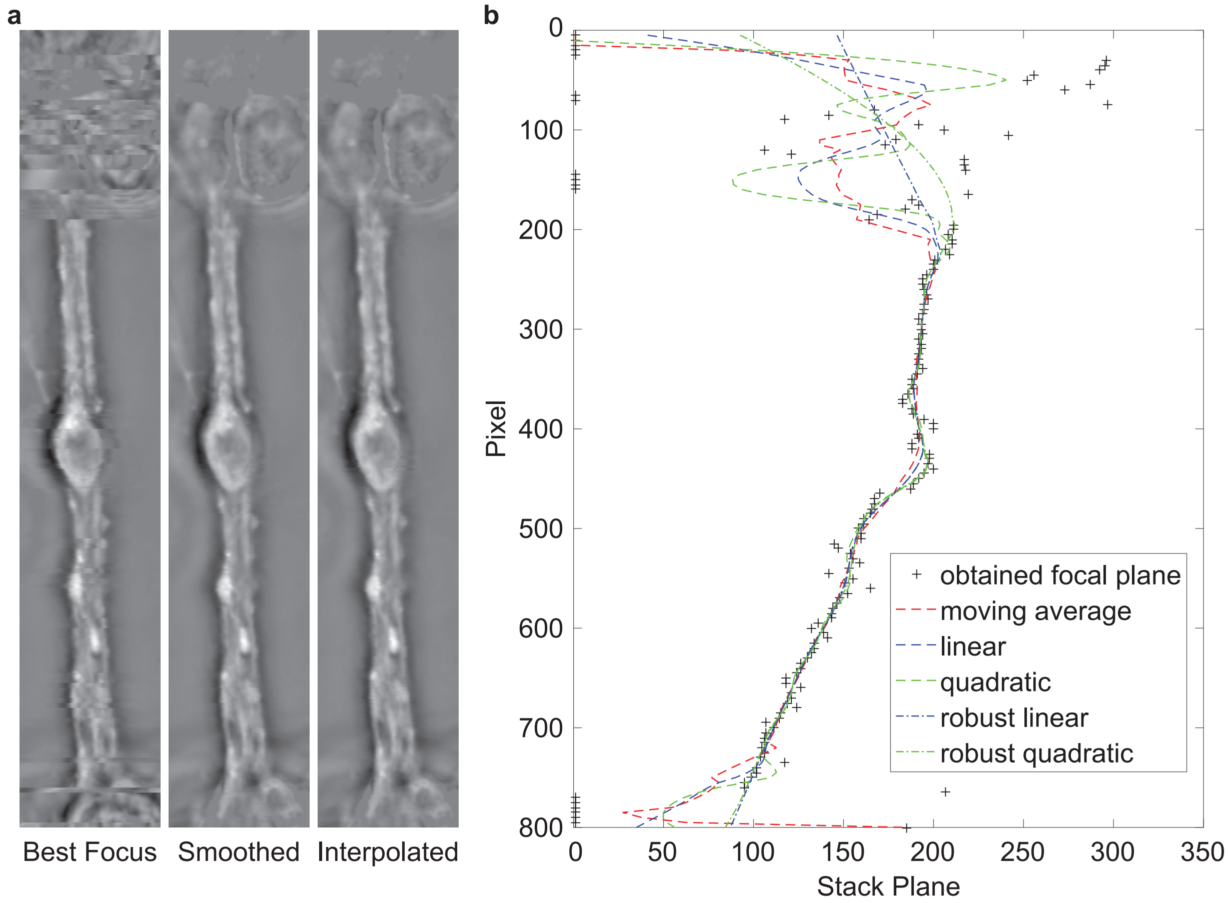

Figure S2: Reconstruction of SLIM stacks into a single image. (a) A computer-determined focal plane among the stack planes is obtained for every "5 pixel × width" section along the y-direction using the maximum gradient method. The reconstructed image (left) is grainy because of the discretized approach. The resulting pixel vs focal plane data is smoothed using the robust linear method. The reconstructed image (center) after smoothing shows significant improvements. This can be further improved (right) by linear interpolation of the 5-pixel data for every pixel. (b) We attempted multiple smoothing algorithm before we finalized on the robust linear method as it preserves feature at the extreme ends of the axon.

Supplementary Figure S3

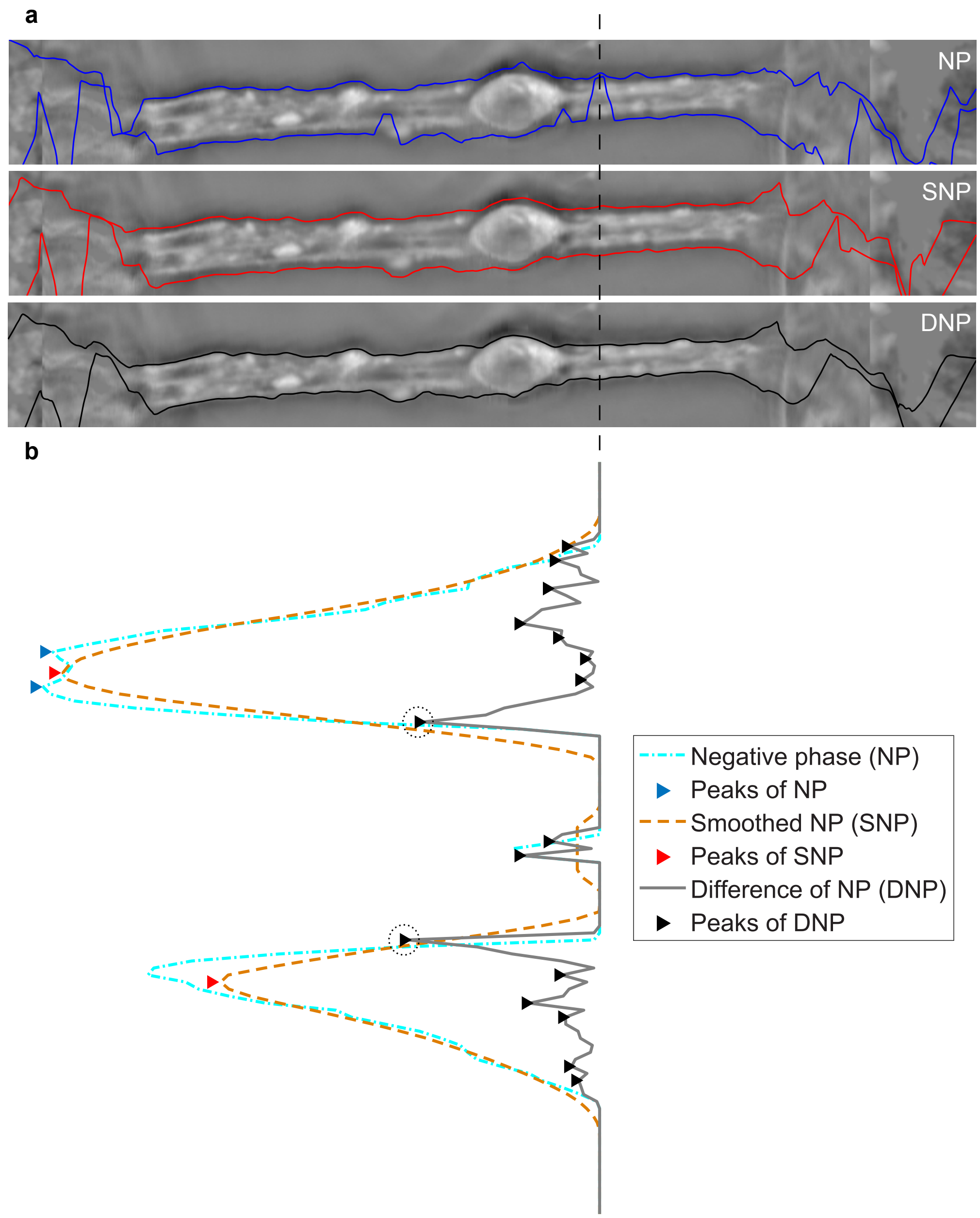

Figure S3: Determination of axon boundary in reconstructed SLIM images. (a) A negative phase region can be observed from the SLIM data at the 2 boundaries of the axon, which we can use to our advantage for diameter determination. We first look for the 2 maximum negative peaks (NP). However, there are local peaks that can bias the results. A moving average smoothing (SNP) takes care of that. The difference curve (DNP) of the smoothed data is obtained to locate the point of maximum slope (similar to taking a 2nd derivative with a continuous function), which we defined as the boundary points. (b) Plot of the negative phase data along the dotted line drawn in (a) to better illustrate the process. The peaks are pointed out in triangles. The final boundary points are circled in dotted line. Note that the phase values reported in the main text was never smoothed; the smoothing is done only during boundary determination.

Supplementary Figure S4

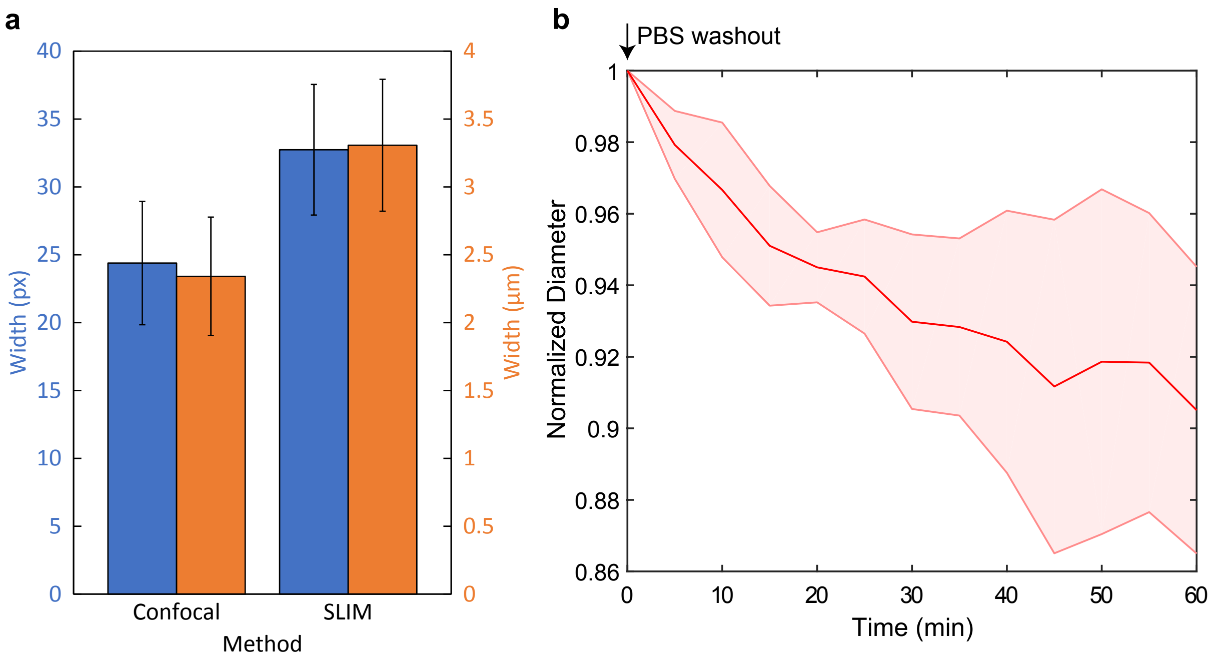

Figure S4: (a) Axon diameter in microns and pixels as measured by confocal and SLIM respectively. (b) Axons incubated under cytoD for 60 minutes before subjected to a pbs washout. Data depict diameter immediately after the washout. All shaded regions indicate error bar in standard deviation.

Supplementary Figure S5

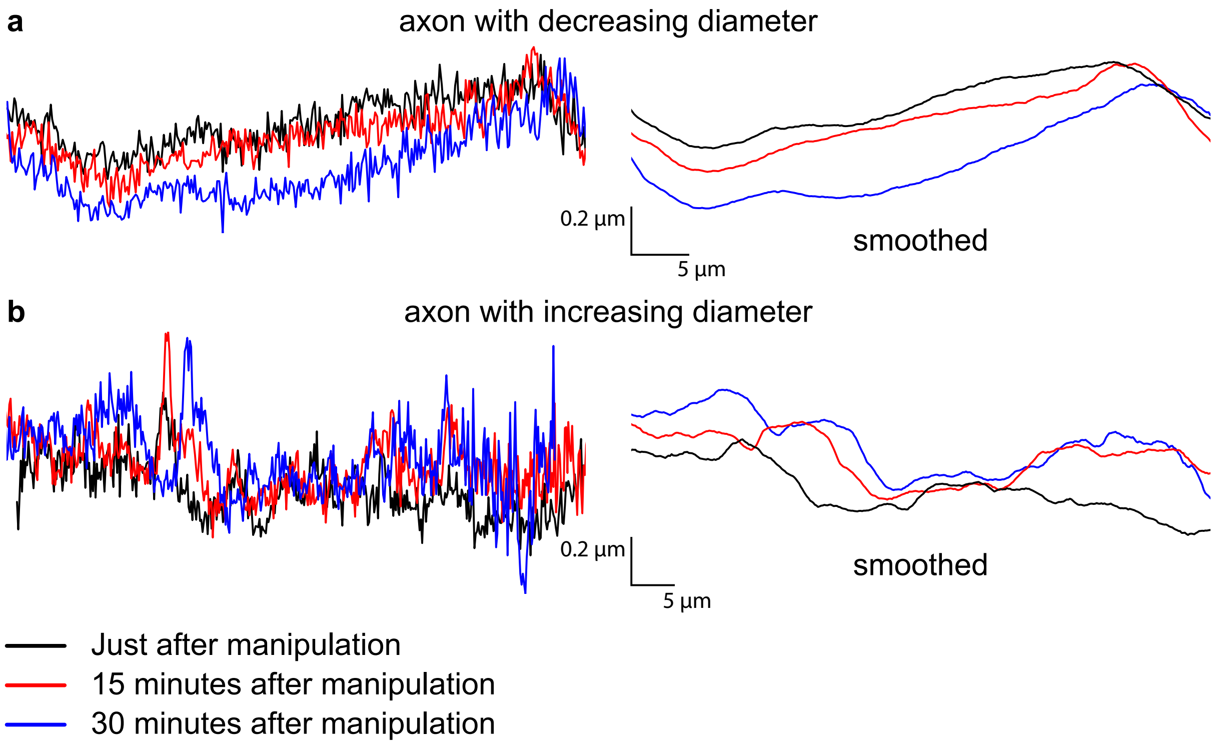

Figure S5: Spatial changes of axon diameter. Sample confocal diameter data of an axon (a) with decreasing diameter and an axon (b) with increasing diameter due to mechanical stretch and cytoD treatment respectively. The plots are colored (given in the legend) according to the time (0, 15, 30 minutes) after the manipulation. Diameter changes in either direction happen along the entire length of the axon. This is better visualized when the raw data is smoothed (right, 50 points moving average). Both axes have units in microns.

Supplementary Figure S6

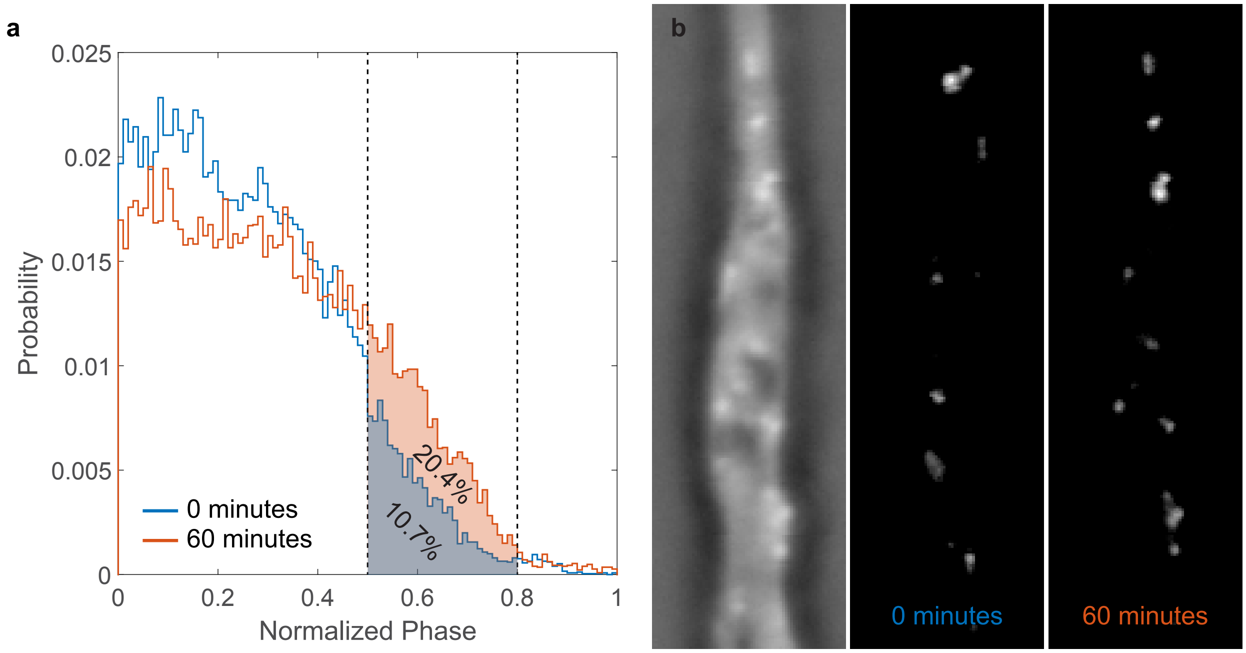

Figure S6: Analysis of axons with phase increase. (a) Phase histogram of axons before (blue) and after (red) cytoD incubation. Pixels are sorted into 100 equal bins according to their normalized phase values $\hat{p} = \frac{p}{p_{max}}$. The y-axis is reported in probability instead of frequency for comparison purposes. Sum of probabilities is equal to 1 in both blue and red curves. Only pixels determined to be inside the axons by the algorithm previously described are used. The 2 dotted lines at $\hat{p} = 0.5$ and 0.8 respectively indicate the range of phase values of the new mass coming in. The total probability of phase values that fall inside the region of interest is reported for both time points. (b) An axon among the group was selected randomly, and only pixels with $0.5 \leq \hat{p} \leq 0.8$ are shown. The resulting structures have vesicle-like morphology.

Supplementary Note

Robustness of average diameter $\langle d \rangle$

Let us consider an in-plane axon positioned at an angle not orthogonal to the x-axis (Fig. S1b). We called the main axis of the axon l-axis. Note that the l-axis does not have to be straight (the axon could be curved). The average diameter computed along the x-axis, i.e. $\langle d \rangle_x$, is:

$$\langle d \rangle_x = \frac{\int_{L_x} d_x(x) dx}{\int_{L_x} dx}, \tag{1}$$

where L_x denotes the limits of the projected length of the axon on the x-axis. $d_x(x)$ is the distance between the 2 edges (assuming we have a way to compute that) of the axon along the x-direction. We are however interested in the average diameter computed along the l-axis, i.e. $\langle d \rangle_l$, instead:

$$\langle d \rangle_l = \frac{\int_{L_l} d_l(l) dl}{\int_{L_l} dl}, \tag{2}$$

where L_l denotes the limits of the length of the axon on the l-axis. $d_l(l)$ is the distance between the 2 edges of the axon parametrized in l and m with $m \cdot l = 0$ and $m \cdot z = 0$. Notice that the numerator in Eq. 1 & 2 is equivalent, i.e.:

$$\int_{L_x} d_x(x) dx = \int_{L_l} d_l(l) dl, \tag{3}$$

because they are both describing the area of the axon, assuming the edge effect is small at the limits. The difference lies in the denominator, in which

$$\int_{L_x} dx = \int_{L_l} cos(\theta(l)) dl, \tag{4}$$

where θ is the angle between the 2 running axes.

In most axon, θ does not depend on l because of the intrinsic tension that would straighten the axon. And therefore Eq. 4 becomes:

$$\int_{L_x} dx = cos(\theta) \int_{L_l} dl. \tag{5}$$

From Eq. 1, 2, 3, & 5

$$\langle d \rangle_l = \langle d \rangle_x cos(\theta). \tag{6}$$

We can then arrive to the conclusion that the average diameter is affected by the orientation of the axon simply by a factor of $cos(\theta)$. This facilitates batch processing particularly if the axon is moving during time-lapse imaging. In cases that the axon is not straight, we compute the path length $\int_{L_l} dl$.
